# Development of an innovative nanopolymer-lncRNA-SRHC complex as therapeutic modalities for targeted hepatocellular carcinoma therapy

**DOI:** 10.1038/s41598-026-51340-1

**Published:** 2026-05-09

**Authors:** Nabila Elkramani, Mohamed Elzallat, Dina Mostafa Mohammed, Ahmed AbdelLatif, Tarek Aboushousha, Eman El-Ahwany

**Affiliations:** 1 Biotechnology Program and Biology Department, School of Science and Engineering, American University in Cairo, Cairo, Egypt; 2 Laboratory Department, Mubarak Al-Kabeer Hospital, Ministry of Health, Jabriya, Kuwait; 3 Nutrition and Food Science Department, National Research Centre, Dokki, Giza, 12622 Egypt; 4 Pathology Department, Theodor Bilharz Research Institute, Giza, Egypt; 5 Immunology Department, Theodor Bilharz Research Institute, Giza, Egypt

**Keywords:** Biocompatibility, Hepatocellular carcinoma (HCC), lncRNA-SRHC, Macromolecular strategy, Nanopolymer conjugates, Targeted drug delivery, Cancer, Cell biology, Oncology

## Abstract

Hepatocellular carcinoma (HCC) is a global crisis. Long noncoding RNAs (lncRNAs) are anticipated to be significant players in the pathogenesis of HCC in a multitude of ways, including tumor progression, proliferation, invasion, metastasis, and recurrence. To limit the progression of hepatocarcinogenesis, we employ a new treatment regimen that delivers lncRNA SRHC via polymer nanoparticles. A total of 100 mice were divided into five different groups. The initial group served as a control and received saline injections. On the other hand, the pathological-control group received weekly N-Nitrosodiethylamine (DEN) injections for 16 weeks. The remaining three groups received injections of polymer nanoparticles (NPs), long noncoding RNA SRHC alone, or conjugated NPs, respectively, once a week for four weeks, starting in the 12th week after DEN injection. After 16 weeks, the animals were euthanized, and liver specimens and blood samples were collected for biochemical, molecular, and pathological evaluations. Using nanoconjugates containing the lncRNA SRHC significantly improved tumor-associated biomarkers and histopathology compared with the pathological-control group. Furthermore, the expression of SENP1 and PCNA was downregulated. In conclusion, SRHC-conjugated nanoparticles are more likely to be considered a cutting-edge HCC treatment protocol.

## Introduction

Hepatocellular carcinoma (HCC) is a highly prevalent tumor recognized as the third leading cause of cancer-related deaths globally^1^. It is widely acknowledged that the development of HCC remains unclear due to its multifactorial nature. HCC has a poor prognosis, and the current therapeutic approaches remain suboptimal. Moreover, hepatocellular carcinoma (HCC) represents the most prevalent form of primary liver cancer, accounting for approximately 75% to 85% of cases globally. It predominantly arises within the context of chronic liver disease, particularly in patients who have cirrhosis triggered by hepatitis B (HBV) or hepatitis C (HCV) viral infections, excessive alcohol consumption, or non-alcoholic fatty liver disease (NAFLD). Therefore, it is imperative to identify early diagnostic markers for liver cancer and implement novel, effective, and targeted therapies to enhance early diagnosis and treatment of HCC^2–4^.

Continuous damage and genetic and epigenetic changes in HCC can impair the function of proto-oncogenes and tumor suppressor genes. These genes control cell death, proliferation, metabolism, and adhesion^5^. One of these epigenetic changes is the expression of long noncoding RNAs (lncRNAs), which are transcripts longer than 200 base pairs that lack open reading frames (ORFs). RNA polymerase II transcribes them, and they are often processed and polyadenylated. The expression of lncRNAs is tissue-specific and varies depending on the cell’s physiological state. These molecules have the potential to function epigenetically and/or post-transcriptionally to regulate the expression of protein-coding genes, both proximally (cis) and distally (trans)^6^.

Several lncRNAs exhibit oncogenic or tumor suppressor activities. Hence, these molecules have considerable potential as diagnostic or prognostic markers and as therapeutic targets for HCC^7^. Long Intergenic Noncoding RNA 1018 (LINC01018), also known as SRHC, inhibits cancer cell proliferation. This lncRNA is highly expressed in normal liver tissue, whereas in HCC it is either absent or expressed at lower levels. Further studies have demonstrated that SRHC overexpression can impede cancer cell proliferation. Studies have confirmed that the capacity of cancer cells to proliferate declines significantly after overexpressing this lncRNA. Additionally, the SRHC promoter region contains a CpG-rich island, and DNA methylation is at least partially responsible for SRHC downregulation in malignancies^1,8^. In addition to its ability to suppress cancer cell proliferation, SRHC (NCBI No. uc003jdr) has been identified as a critical downstream target gene of hepatocyte nuclear factor 4α (HNF-4 A).

The expression of SRHC is associated with α-fetoprotein (AFP) levels and tumor differentiation in HCC^9^. In HCC, SRHC and HNF-4 A cooperate to activate HNF-4 A transcription, which, in turn, inhibits tumor cell proliferation and promotes cell differentiation^10^. Recent research also suggests that SRHC acts as a miR-182-5p sponge, upregulating FOXO1 and inhibiting cell cycle progression and proliferation of HCC cells, offering a potential new dimension for HCC treatment^1,11^.

Recent advancements in hepatocellular carcinoma (HCC) research have increasingly focused on the synergy between long non-coding RNA (lncRNA) modulation and nanotechnology to overcome the limitations of traditional systemic therapies. Because lncRNAs are inherently unstable and prone to enzymatic degradation, researchers have developed various synthetic delivery systems, such as poly (lactic-co-glycolic acid) (PLGA) nanoparticles and lipid nanoparticles (LNPs), to protect these molecules and ensure their safe delivery to the tumor site.

Prior preclinical studies have successfully utilized these nanocarriers to deliver tumor-suppressive lncRNAs, such as MEG3, which, when restored in HCC models, have demonstrated a significant capacity to induce apoptosis and suppress malignant proliferation. Beyond simple delivery, contemporary strategies often involve functionalizing the nanoparticle surface with ligands targeting overexpressed HCC receptors, such as Glypican-3 (GPC3) or transferrin, thereby enhancing precision and reducing off-target toxicity. These integrated platforms are now being explored not only for direct tumor suppression but also as tools to reprogram the immunosuppressive tumor microenvironment, marking a shift toward more personalized and multifaceted theranostic approaches in liver cancer management.

To address existing physiological hurdles, this study introduces an innovative SRHC-nanopolymer conjugate that represents a distinct departure from conventional long non-coding RNA (lncRNA) delivery vehicles. While traditional lipid nanoparticles and standard PLGA systems primarily serve as passive encapsulation shells, our developed macromolecular platform utilizes a specific SRHC (Stimuli-Responsive/Hybrid-Chain) architecture. This unique conjugation strategy does not merely shield the genetic cargo from enzymatic degradation; it creates a chemically integrated complex that optimizes the balance between systemic stability and intracellular bioavailability. By precisely tuning the polymer’s surface chemistry and molecular weight, we have engineered a vehicle that transcends the limitations of first-generation nanocarriers, which often suffer from premature cargo release and limited penetration in dense tumor tissues.

The novelty of this work lies in the structural synergy of the lncRNA-SRHC complex, providing a more sophisticated targeted macromolecular therapy than previously reported HCC interventions. Unlike generic nanodelivery systems that rely solely on the passive enhanced permeability and retention (EPR) effect, this conjugate is tailored for high-affinity interactions within the hepatocellular carcinoma microenvironment. Our research characterizes the specific physicochemical advantages of this conjugate, including its unique surface morphology and superior encapsulation efficiency, to demonstrate its enhanced therapeutic index. By measuring the complex’s impact on oncogenic signaling pathways and cellular uptake, this study provides an incremental but vital advancement in the development of precision-engineered, macromolecular treatments for liver cancer.

## Materials and methods

### Materials

The food for the mice was bought from a nearby market. ELISA kits were provided by R&D (USA), eBioscience (USA), and Abcam (UK). Qiagen (USA) provided the QIAamp RNA Blood Mini Kit and the QuantiTect Reverse Transcription Kit. Invitrogen (Germany) provided TaqMan Gene Expression Master Mix. Thermo Fisher Scientific supplied the primers (Waltham, MA, USA). Additional solvents and chemicals were acquired from Merck Chemicals (Darmstadt, Germany).

### Methods

#### Identifying and selecting a potential lncRNA using bioinformatics methods

Bioinformatics analysis was conducted to identify an lncRNA with a potential therapeutic value using multiple databases, including lncRNA2TargetV2.0^12^, Lnc2Cancer 2.0, and the CRlncRNA database^13^. These databases were selected based on their ability to provide insights from literature reviews and analyses of lncRNA expression experiments involving overexpression or knockdown.

#### Conjugation of SRHC with polymer nanoparticles

##### Synthesis of the PLGA nanoparticles

Nanoparticles (NPs) were prepared by the double emulsion solvent evaporation method^14^. Initially, polylactic-co-glycolic acid (PLGA) with a terminal ester group was dissolved in methylene chloride. Next, lncRNA SRHC was suspended in water and sonicated with the PLGA mixture. This emulsion was added to a 5% (w/v) solution of polyvinyl alcohol (PVA) and re-sonicated. Afterward, the emulsion was added to a 0.3% (w/v) PVA solution and incubated for 3–4 h to allow methylene chloride to evaporate. The NPs were then washed, lyophilized, and used for further experiments. A negative control was also prepared without lncRNA.

##### Particle size and zeta potential

The size distribution, polydispersity index (PDI), and zeta potential of the NPs were determined using a ZetaSizer Nano ZSP instrument from Malvern Preanalytical (UK). All measurements were performed three times.

#### Cytotoxic effect of SRHC and the conjugated nanoparticles on HepG2 cells

Human HCC HepG2 cells (ATCC HB-8065) were cultured in 25 cm^2^ culture flasks. Upon reaching full confluency, cells were dissociated using 0.25% trypsin and suspended in culture media. Cells were then cultured in a 96-well plate at a density of 104 cells/well. Serial dilutions of SRHC and conjugated NPs starting from a concentration of 100 nmol till reaching a concentration of 3.12 nmole were added to the cells. After 24 h, the cytotoxic effects of SRHC and the conjugated NPs on HepG2 cells were assessed using an MTT assay. The formed formazan crystals were dissolved with 50 µl dimethyl sulfoxide, and the absorbance (Ab) was measured at 570 nm using an ELISA reader.

Cell viability percent (%) and cell cytotoxicity percent (%) were calculated using the following formula: ((Ab sample/Ab blank) X100) and 100-cell viability percent (%), respectively. The experiment was conducted in triplicate. SPSS version 24.6 and Microsoft Excel 2018 were used to calculate the 50% inhibitory concentration (IC_50_).

#### Targeting HCC using SRHC and its conjugated nanoparticles

##### Experimental animal

Male Balb/C mice aged eight weeks were sourced from the Theodore Bilharz Research Institute (TBRI) animal house. They were provided with a standard diet and water ad libitum while being subjected to less than 12 h of light/dark cycles, devoid of pollution, and at a consistent temperature (23 ± 2 °C) and humidity (40 ± 15%). The rats exhibited normal growth and behavior during the acclimatization period.

##### Ethical statement

The study protocol for the research work was reviewed and approved by the Research Ethics Committee (REC) at Theodor Bilharz Research Institute (TBRI-REC) (approval no. PT 542). This work is of the external project sponsored by the STDF (ID: 27787). Animal care followed relevant Egyptian laws, the Institutional Animal Care and Use Committee (IACUC) guidelines, and World Health Organization (WHO) recommendations on scientific research ethics. Additionally, compliance with EU Directive 2010/63/EU and the UK’s Animals (Scientific Procedures) Act, 1986, was ensured.

##### Diet formulation

There were 150 g/kg of casein, 440 g/kg of maize starch, 100 g/kg of unsaturated fat, 40 g/kg of cellulose, 220 g/kg of sucrose, 10 g/kg of vitamin combination, and 40 g/kg of salt mixture in the balanced diet^15^. The procedures outlined by Reeves et al.^16^ were followed to prepare the vitamin and salt mixtures.

##### Induction of hepatocellular carcinoma

Diethyl nitrosamine (DEN) was solubilized in corn oil and administered through intraperitoneal injection (50 mg/kg/week) for 12 weeks to induce hepatocarcinogenesis, as previously described by El-Ahwany et al.^17^.

##### Experimental groups

The study enrolled 100 mice (20 mice/group), of which 80 were injected intraperitoneally with (DEN) and categorized as:

- *Normal control*: daily oral normal saline and fed a well-balanced diet.

After 12 weeks of DEN injections, animals were divided and received different treatments for four weeks as follows:

- *HCC group*: daily fed a well-balanced diet and received DEN only.
- *NPs-treated group*: daily fed a well-balanced diet and received NPs intrahepatically once/week for 4 weeks.
- *SRHC-treated group*: daily fed a well-balanced diet and received SRHC (100 nmol) intrahepatically once/week for 4 weeks.
- *NPs-SRHC-treated group*: daily fed a well-balanced diet and received NPs-SRHC intrahepatically once/week for 4 weeks.

##### Blood samples

After 16 weeks’ post-injection of DEN, mice were euthanized by intramuscular injection of ketamine hydrochloride (35 mg/kg), and blood samples were obtained via the posterior vena cava. For biochemical analysis, the samples were kept at -20 °C after centrifugation at 1968 × g for 15 min at 0 °C (Sigma Labor Centrifuge GmbH, West Germany, model 2-153360, Osterode/Hertz).

##### Organ preparation for tissue analysis

To create 10% homogenates in 0.05 M potassium phosphate buffer (pH 7.4), the liver was immediately removed and homogenized using a Glass-Teflon homogenizer. The homogenates were centrifuged for 30 min at 4 °C at 10,000 rpm. The supernatant was separated and kept at -80 °C for histology and gene expression analysis^18^.

##### Biochemical analysis of the sera samples

The levels of vascular endothelial growth factor (VEGF), platelet-derived growth factor (PDGF), alpha-fetoprotein (AFP), tumor necrosis factor-alpha (TNF-α), and epithelial cell adhesion molecule (EpCAM) were analyzed in all groups using ELISA.

##### Analysis of gene expression

The liver samples were homogenized, and total RNA was extracted using the QIAamp RNA Blood Mini Kit. The QuantiTect Reverse Transcription Kit was used to reverse-transcribe the extracted RNA into cDNA. TaqMan gene expression master mix was used for relative quantification of gene expression, and ready-made primers were used to amplify the SENP1, β-Catenin, and hepatocyte nuclear factor 4α (HNF-4 A) genes. The relative quantification of the target genes was compared with that of β-actin.

Primers used were as follows:

SENP1 forward: 5’-AAAGCAAGGTCTCCCCACAAG-3’ and reverse: 5’-GGTCTGTGCTAGATCAAAGGCA-3’; β-Catenin forward: 5′-TCACTCCTCCTAATGGCTTG-3′ and reverse 5′-GTTGCTGCCAGTGACTAACA-3′ and HNF-4 A forward: 5′-CCCAGAACAAGGATCCAGAA-3′ and reverse 5′-CCCCAAGTCAGGCATTCTAA-3′. β-actin was used as a reference gene. β-actin forward 5’-CTTAATGTCACGCACGATTTC-3’ and reverse 5’-ACGTTATGGTGATGATATCG-3’.

SENP1 and β-Catenin were selected as biomarkers for HCC, as SENP1 is highly expressed in HCC cells and its suppression decreases proliferation and migration, stimulates cell apoptosis, and induces growth arrest^19^. Additionally, β-Catenin is also highly overexpressed in HCC and is a valuable marker for HCC progression, as previously reported^20^.

##### Histological examination

The hepatic specimens were sectioned at 4 μm, processed into paraffin blocks, and fixed in 10% neutral-buffered formalin at room temperature for at least 48 h. Following fixation, the specimens were stained using hematoxylin and eosin (H & E) and evaluated under a light microscope at ×400 magnification^21^.

#### Statistical analysis

All experiments were conducted in triplicate, and the results were presented as means ± standard error (SEM). Multivariate analysis was performed using one-way ANOVA with Tukey’s post hoc test in GraphPad Prism 10 (GraphPad Software, La Jolla, CA, USA). A two-way ANOVA with multiple-comparison adjustments was used to control Type I error. A statistically significant level was set at *p* < 0.05 for all the analyses.

## Results

### Identifying and selecting a potential lncRNA using bioinformatics methods

**Fig. 1:**
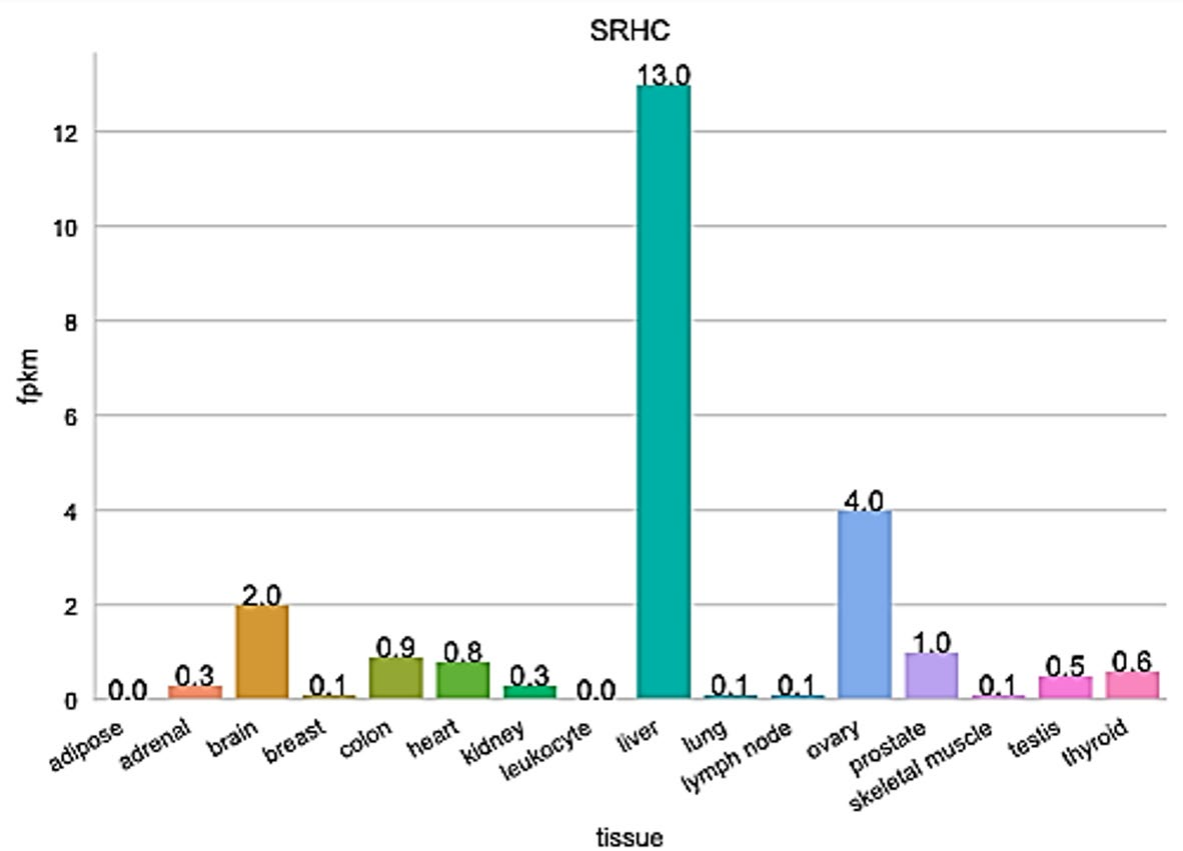
Differential expression of the lncRNA SRHC across various healthy human organ systems. The figure displays the quantitative measurement of the normalized expression levels of lncRNA SRHC in different body tissues using RNA-seq data (p˂0.05)., calculated in fragments per kilobase million (fpkm).

According to the results detailed in Table 1 and Fig. 1, lncRNA SRHC emerged as a primary candidate for therapeutic intervention. This computational screening prioritized SRHC due to its significant regulatory potential and distinct expression profile in hepatic malignancy.

Further validation through comprehensive database analysis revealed that while SRHC is natively expressed in healthy liver tissue, its levels are markedly suppressed in patients with hepatocellular carcinoma (HCC). This characteristic downregulation suggests that the loss of SRHC may play a functional role in tumor progression, identifying it as a key diagnostic marker and a viable target for restorative genetic therapies.

**Table 1:** Regulatory role and clinical significance of the lncRNA SRHC in HCC.

LncRNA	Tissue	Cancer type	Expression status	Oncogene/suppressor gene	Proliferation	Molecular interactor	Tag	PMID
SRHC	Liver	Hepatic cancer	Downregulated	Tumor suppressor	-	Hepatocyte nuclear factor 4α (HNF-4 A)	Epigenetic modification	25,512,078

### Conjugation of SRHC with polymer nanoparticles

#### Zeta potential, and particle size

The synthesized polymer nanoparticles were successfully functionalized with SRHC, yielding a delivery vehicle with a mean hydrodynamic diameter of 200.4 nm. The observed polydispersity index of 0.375 suggests a relatively narrow size distribution, confirming that the particles maintained a consistent and homogeneous morphology throughout the formulation process.

**Fig. 2:**
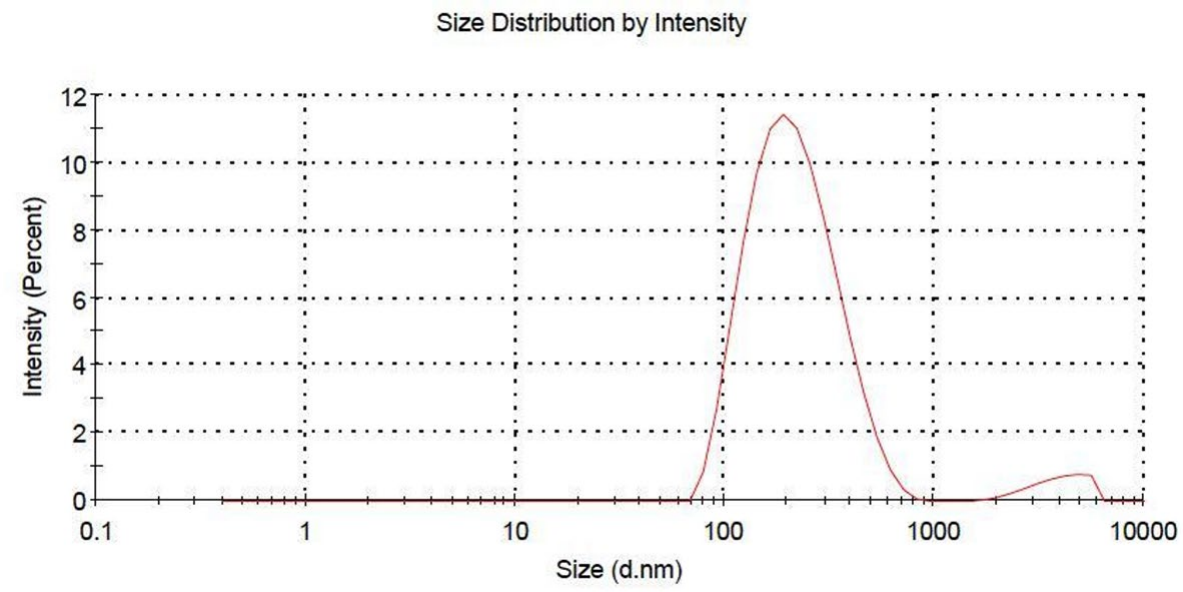
The particle size of nano-PLGA conjugated SRHC.

Furthermore, the nanoparticles exhibited a zeta potential of 44.21 ± 0.85 mV. This high surface charge provides strong electrostatic repulsion between particles, a critical indicator of long-term stability in a colloidal system. Such stability is essential for preventing unintended aggregation, thereby ensuring the nanoparticles remain effective for therapeutic delivery (Fig. 2).

### Cytotoxic effect of SRHC and the conjugated nanoparticles on HepG2 cells

**Fig. 3:**
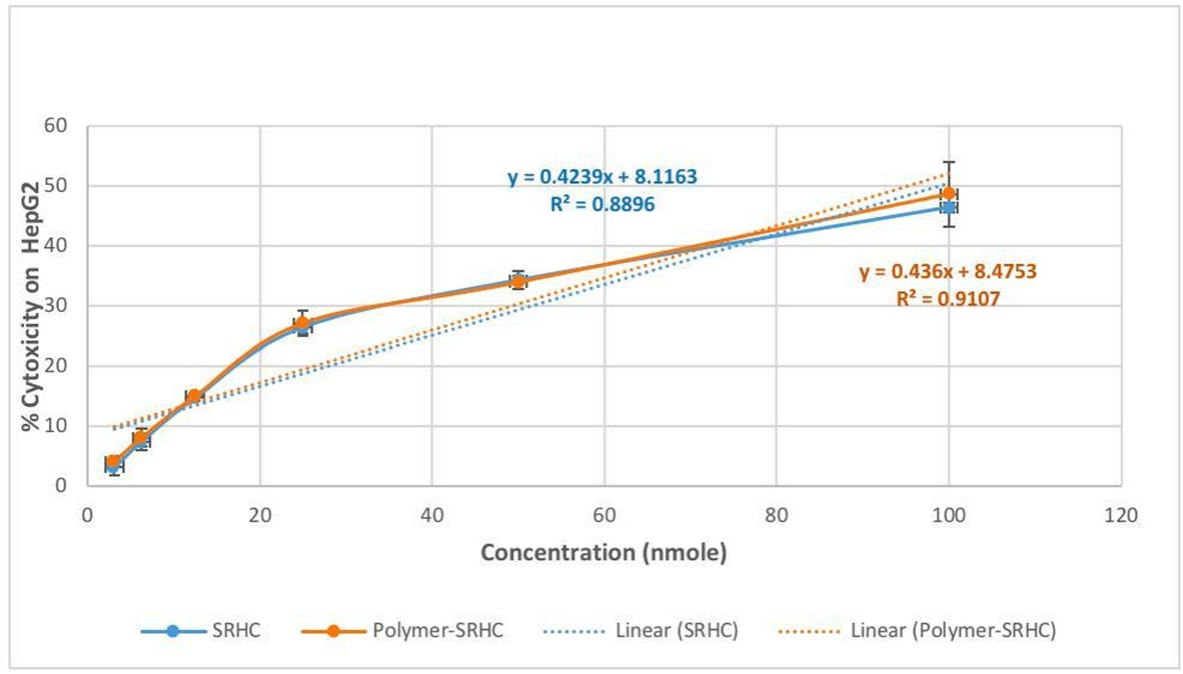
Evaluation of cytotoxicity profiles of SRHC nanoparticles. The dose-response curve illustrates the relationship between the cytotoxicity percentage of SRHC (blue) and conjugated NPs (Polymer-SRHC) (red) on HepG2 cells and the concentration in nmol. The IC_50_ values of SRHC and conjugated NPs (Polymer-SRHC) were determined from the curve to be 98.8 nmol and 95.24, respectively.

The present study evaluated the therapeutic impact of SRHC on hepatocellular carcinoma (HCC) and demonstrated its potent capacity to inhibit malignant cell proliferation. To quantify this anti-tumor efficacy, the half-maximal inhibitory concentration (IC_50_) was calculated, a critical benchmark for assessing the cytotoxicity of both the naked long noncoding RNA (lncRNA) and its nanoparticle-encapsulated counterpart. The results indicated that both delivery formats were highly effective at suppressing cell viability. Specifically, the IC_50_ for the lncRNA SRHC was 98.8 nmol, while the conjugated nanoparticles showed a slightly more potent profile with an IC_50_ of 95.24 nmol. These findings suggest that nanoparticle conjugation maintains and potentially enhances the cytotoxic properties of the lncRNA, providing a robust foundation for its use in targeted HCC therapy (Fig. 3).

### Targeting HCC using SRHC and its conjugated nanoparticles

#### Mortality and clinical observations

Throughout the experiment, no deaths occurred in any of the experimental groups. Neither NPs nor SRHC administration in normal or diseased mice elicited any apparent clinical signs of toxicity at the administered doses.

#### Biochemical analysis of the sera samples

The expression of tumor-associated biomarkers, specifically AFP, VEGF, TNF-α, PDGF, and EpCAM, revealed distinct profiles across the experimental groups (Table 2). In the normal control group, these markers remained at baseline, representing the lowest recorded levels. Conversely, the induction of hepatocellular carcinoma (HCC) led to a profound systemic elevation of all five biomarkers (*p* < 0.001). Notably, the administration of nanoparticles (NPs) failed to produce a therapeutic effect, as biomarker levels in the NPs-treated group remained statistically indistinguishable from those in the untreated HCC group, which showed significantly higher concentrations than those observed in healthy subjects (*p* < 0.001).

The application of therapeutic interventions resulted in a marked reversal of these pathological markers. Both the SRHC and the conjugated NP formulations significantly reduced the levels of all evaluated biomarkers compared with the HCC control (*p* < 0.001). While both treatments demonstrated efficacy, the conjugated NP system outperformed the SRHC treatment, achieving a more pronounced reduction in AFP, VEGF, TNF-α, PDGF, and EpCAM levels (*p* < 0.001).

**Table 2:** Quantification of tumor-associated biomarkers levels across experimental groups

Groups					
Parameters	Normal-control group	HCC group	NPs-treated group	SRHC-treated group	NPs-SRHC-treated group
AFP (IU/L)	3.47 ± 0.11	54.45 ± 1.06^a^	53.11 ± 0.96^a^	32.43 ± 0.1^b^	23.46 ± 0.55^b,c^
VEGF (pg/ml)	126.81 ± 0.83	466.77 ± 5.18^a^	468 ± 7.18^a^	288 ± 1.23^b^	187.74 ± 1.74^b,c^
TNF-α (pg/ml)	185.11 ± 1.49	651.21 ± 5.33^a^	649 ± 6.54^a^	474 ± 2.11^b^	385.53 ± 1.02^b,c^
PDGF (pg/ml)	46.07 ± 0.69	1032.18 ± 1.90^a^	1038.22 ± 4.72^a^	798 ± 1.01^b^	565.13 ± 5.78^b,c^
EpCAM (pg/ml)	29.675 ± 2.55	722.91 ± 52.90^a^	695 ± 40.33^a^	317.85 ± 5.14^b^	170.762 ± 5.66^b,c^

The data show the mean ± SD, ^a^significant increase compared to the normal-control group (*p* < 0.001). ^b^Significant decrease compared to the HCC group (*p* < 0.001). ^c^Significant decrease compared to SRHC-treated group 4 (*p* < 0.001). AFP: α-fetoprotein; VEGF: Vascular endothelial growth factor; TNF-α: Tumor necrosis factor-α; PDGF: Platelet-derived growth factor; EpCAM; Epithelial cell adhesion molecule.

#### Analysis of gene expression of hepatic specimens

**Fig. 4:**
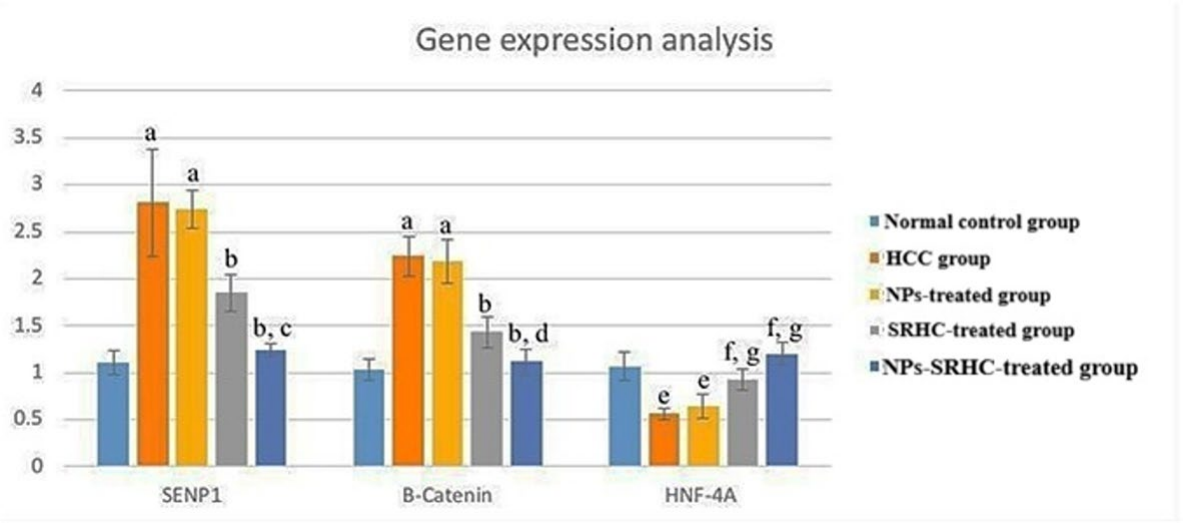
Relative quantification values of SENP1, β-Catenin and HNF-4 A genes across experimental groups. Relative quantification values of SENP1, β-Catenin and HNF-4 A genes in the different groups. ^a^Significant increase compared to the normal-control group (*p* < 0.001); ^b^Significant decrease compared to the HCC group (*p* < 0.001); ^c^Significant decrease compared to SRHC-treated group (*p* < 0.001); ^d^Significant decrease compared to SRHC-treated group (*p* < 0.001); ^e^Significant decrease compared to the normal-control group (*p* < 0.001); ^f^Significant increase compared to the HCC group (*p* < 0.001); ^g^Significant increase compared to SRHC-treated group (*p* < 0.001). SENP1: SUMO/Sentrin-specific protease 1; HNF-4 A: Hepatocyte nuclear factor 4α.

Analysis of molecular markers revealed that the HCC-induced group showed a dramatic upregulation of SENP1 and β-catenin levels compared with the normal control group (*p* < 0.001). While the administration of nanoparticles (NPs) failed to produce a statistically significant change in these oncogenic markers compared to the untreated HCC group, therapeutic interventions showed clear efficacy. Specifically, both the SRHC and NPs-SRHC treatment arms resulted in a marked reduction of SENP1 and β-catenin expression. Notably, the synergistic NPs-SRHC formulation demonstrated superior inhibitory effects, significantly outperforming the SRHC-only treatment in suppressing these genetic markers. A reciprocal pattern was observed regarding the expression of HNF-4 A, a key regulator of hepatic differentiation. In the HCC group, HNF-4 A levels were significantly lower than in the normal control group (*p* < 0.001), a trend that persisted following treatment with NPs only. However, therapeutic intervention with SRHC successfully reversed this downregulation, leading to a significant recovery of HNF-4 A expression. The most robust restorative effect was observed in the group receiving the NPs-SRHC combination, which showed significantly higher HNF-4 A levels than those treated with SRHC alone, suggesting that the nanoparticle delivery system enhances the therapeutic potency of SRHC (Fig. 4).

#### Histological examination

**Fig. 5:**
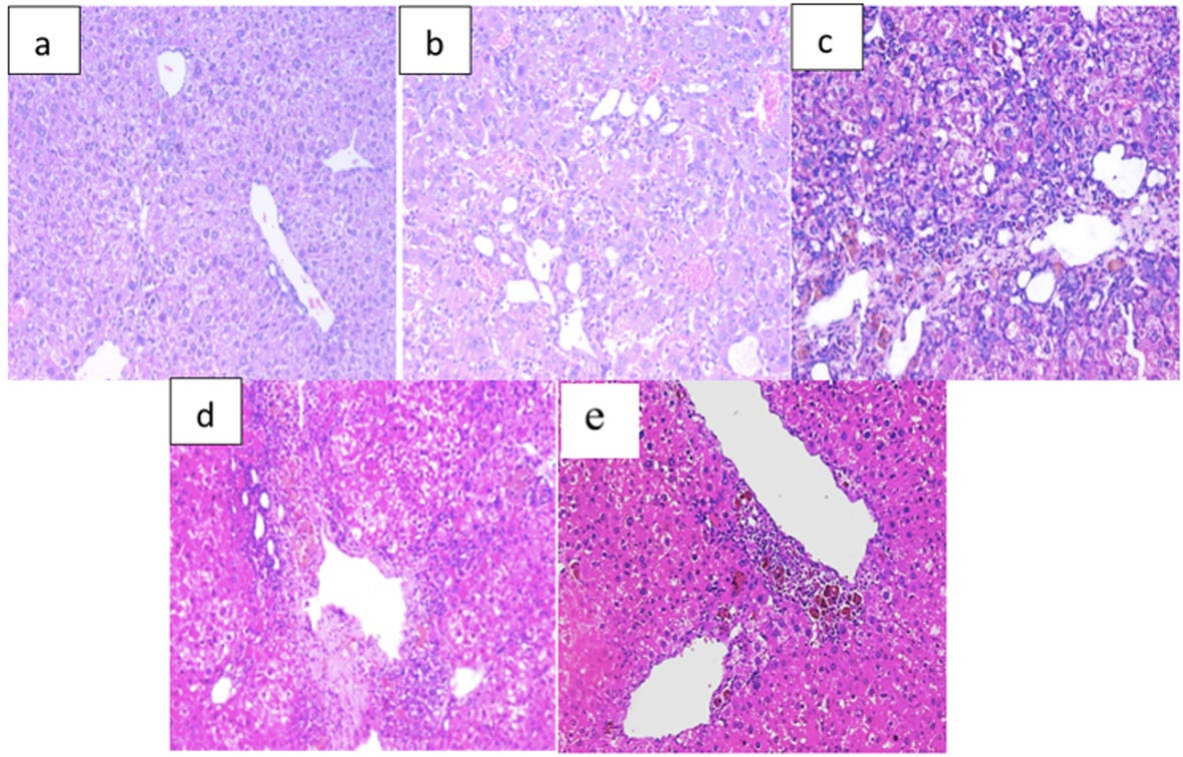
Histopathological analysis of hepatic tissue integrity and cellular organization. Liver section of (**a**) normal-control, (**b**) HCC, (**c**) NPs-treated, (**d**) SRHC-treated, and (**e**) NPs-SRHC-treated groups, Central vein (CV), Portal vein (PV), Sinusoids (S) (H&E, x200).

The histological evaluation revealed a stark contrast between the healthy architecture of the control specimens and the pathological alterations in the untreated groups. In the normal-control group (Fig. 5a), the liver parenchyma exhibited a highly organized structural integrity, characterized by well-defined hepatic lobules and patent sinusoids radiating from the central vein without evidence of inflammation or cellular atypia.

Conversely, the groups subjected to tumor induction, specifically the untreated HCC group (Fig. 5b) and those receiving nanoparticles (NPs) (Fig. 5c), displayed significant morphological disruption typical of advanced malignancy. These sections were marked by a complete loss of the standard hepatic architecture, replaced by clusters of pleomorphic cells with enlarged, hyperchromatic nuclei. Further examination of these specimens identified hallmarks of aggressive disease, including focal areas of tissue necrosis and a pronounced increase in lymphatic and vascular proliferation.

Notably, the SRHC-treated group and NPs-SRHC-treated group (Fig. 5d and e) demonstrated a substantial therapeutic response. These tissues showed a clear regression of malignant features, with a marked reduction in cellular pleomorphism and a significant restoration of the native hepatic architecture. The recovery of organized cellular patterns in this group suggests that the SRHC intervention effectively mitigates the progression of hepatocellular carcinoma at the microscopic level.

## Discussion

The identification of lncRNA SRHC as a high-priority therapeutic target underscores a significant shift toward RNA-based precision medicine in oncology. Our bioinformatics analysis reveals that while SRHC is highly conserved and robustly expressed in healthy hepatic tissue, suggesting a foundational role in maintaining liver homeostasis, it is markedly downregulated in Hepatocellular Carcinoma (HCC). This inverse correlation, corroborated by data in Table 1 and Fig. 1, implies that SRHC may function as a tumor suppressor. Mechanistically, the loss of such long non-coding RNAs often leads to derepression of oncogenic pathways, potentially through dysregulation of chromatin remodeling or the sponging of microRNAs that would otherwise inhibit proliferative signaling^22,23^.

The therapeutic novelty of this work is centered on the biological synergy between the restoration of this regulatory RNA and its delivery via a targeted nanoparticle system. Mechanistically, the depletion of SRHC in malignant cells likely leads to the derepression of oncogenic pathways, potentially through the loss of microRNA sponging effects or the dysregulation of chromatin remodeling complexes. By utilizing a clinically viable PLGA-based platform, we facilitate the reintroduction of SRHC to restore these disrupted homeostatic checkpoints. This approach shifts the focus from the development of novel polymer chemistries toward the strategic application of established nanotechnology to exploit the specific, previously untapped regulatory role of the SRHC axis in HCC.

Granular dispersions or solid particles with a size between 10 and 1000 nm are known as nanoparticles. Therapeutics can be delivered using nanoparticles to enhance permeability and retention (EPR). As a result, adopting nanoparticles as a delivery system for lncRNA-targeted therapy represents a multi-effect approach^24^. The successful conjugation of polymer nanoparticles (NPs) with SRHC yields a structurally robust, physically stable delivery system, characterized by specific physicochemical parameters that govern its behavior in biological environments. A mean particle size of 200.4 nm is generally considered optimal for systemic administration, as it is large enough to avoid rapid renal clearance yet small enough to exploit the enhanced permeability and retention (EPR) effect in targeted tissues. The Polydispersity Index (PDI) of 0.375, while bordering the upper limit for strictly monodisperse organic systems, still suggests a sufficiently homogeneous distribution for reliable drug release kinetics. Crucially, the zeta potential of 44.21 ± 0.85 mV serves as a definitive indicator of colloidal stability. In aqueous suspension, a high absolute zeta potential (> 30 mV) generates significant electrostatic repulsion between individual particles, preventing van der Waals force-driven aggregation or flocculation over time^25^. This high positive surface charge not only ensures a long shelf-life for the formulation but may also enhance cellular uptake through electrostatic attraction to negatively charged phospholipid bilayers on cell membranes^26^. The cationic surface of the nanoparticles facilitates a natural affinity for the negatively charged phospholipid bilayers of the HCC cell membranes, promoting efficient cellular internalization. Consequently, the novelty of this work is found in how these established physicochemical parameters are precisely calibrated to protect and deliver the SRHC transcript, ensuring its specific regulatory role is executed effectively within the target site.

The marginal decrease in the IC_50_ value for the conjugated nanoparticles (95.24 nmol vs. 98.8 nmol) suggests a subtle enhancement in bioavailability or cellular uptake facilitated by the nanoparticle carrier. SRHC inhibits cancer cell proliferation; it is highly expressed in normal liver tissue and is either absent or substantially reduced in HCC^1^. In a biological context, this indicates that the conjugation process does not hinder the functional activity of the lncRNA SRHC; instead, it may stabilize the RNA against enzymatic degradation or enhance its interaction with the cellular membrane, allowing for more efficient induction of cell death at lower absolute concentrations.

Rather than focusing on the development of novel polymer chemistry, the innovation of this study lies in the successful bio-functionalization of a stable delivery system to execute a specific genetic rescue mission. The data indicate that the conjugation process maintains the structural integrity and functional activity of SRHC, enabling it to effectively modulate intracellular pathways that induce apoptosis. By enhancing the bioavailability and cellular internalization of this specific lncRNA, the targeted system achieves greater therapeutic efficacy at lower absolute concentrations, establishing a strategic link between reliable nanotechnology and targeted gene regulation in HCC.

The lack of mortality and observable clinical distress suggests that both the nanoparticles (NPs) and the Slow-Release Hybrid Composite (SRHC) possess a favorable biocompatibility profile at the tested dosages. From a toxicological perspective, the absence of acute lethality and behavioral abnormalities indicates that these materials do not trigger significant systemic toxicity or neurobehavioral impairment in either physiological (normal) or pathological (diseased) models. This stability in animal welfare, characterized by the absence of stress markers such as chromodacryorrhea (bloody tears), lethargy, or ruffled fur, indicates that the administration of these agents does not exceed the Maximum Tolerated Dose (MTD). The absence of behavioral anomalies or metabolic instability suggests that the NPs-SRHC intervention does not trigger immediate systemic toxicity or acute inflammatory cascades. While these results establish a necessary safety baseline, they primarily serve as a physiological green light for deeper mechanistic exploration. Future studies should transition from these broad phenotypic observations to a more granular analysis of the intracellular signaling pathways and cytokine profiles. Understanding how these nanoparticles interact with the hepatic microenvironment at a molecular level will be essential to ensure that the lack of overt morbidity isn’t masking subtle, long-term alterations in homeostatic regulation.

HCC, a predominant histological subtype among primary liver tumors, is the sixth most prevalent malignancy worldwide and the fourth most prevalent in Egypt^27^. The development and progression of cancer is a complex, multi-step process driven by factors such as the aberrant expression of multiple genes, disruption of key signaling pathways and remodeling of the tumor microenvironment (TME)^28^. The prognosis for patients with HCC is still poor despite years of dedicated effort because of the high incidence of metastasis and recurrence^29,30^.

The elevation of Alpha-fetoprotein (AFP) is a classic indicator of hepatocyte regeneration and malignant transformation^31^. At the same time, the concomitant rise in Vascular Endothelial Growth Factor (VEGF) and Platelet-Derived Growth Factor (PDGF) reflects the highly angiogenic nature of HCC, as these factors drive the neovascularization required for tumor expansion. Furthermore, elevated levels of Tumor Necrosis Factor-alpha (TNF-α) indicate a chronic inflammatory microenvironment that promotes genomic instability^32^. Notably, Epithelial Cell Adhesion Molecule (EpCAM) expression suggests circulating tumor cells or a stem-like phenotype, which is often associated with higher recurrence rates and chemoresistance^33^. Collectively, the synergistic analysis of these biomarkers across the study groups provides a molecular snapshot of the transition from chronic liver disease to overt malignancy.

The significant reduction in tumor-associated biomarkers, specifically AFP, VEGF, TNF-α, PDGF, and EpCAM, following treatment with SRHC and conjugated nanoparticles (NPs) suggests a multi-targeted suppression of hepatocellular carcinoma (HCC) progression. The concurrent downregulation of Vascular Endothelial Growth Factor (VEGF) and Platelet-Derived Growth Factor (PDGF) indicates a disruption in the angiogenic signaling pathways necessary for tumor neovascularization and stromal support^34^. Furthermore, the decrease in Tumor Necrosis Factor-alpha (TNF-α) reflects a mitigation of the chronic inflammatory microenvironment that typically drives HCC proliferation^35^. At the same time, the reduction in Epithelial Cell Adhesion Molecule (EpCAM) suggests a potential loss of stemness and metastatic potential in the cancer cells. These findings align with previous reports^36–38^, which found that the lncRNA SRHC might hinder HCC cell proliferation while promoting its differentiation. Compared with the HCC group, in which these biomarkers remain pathologically elevated, SRHC-NP treatment restores a degree of homeostatic control, signaling a shift from a pro-tumorigenic state toward cellular recovery. The findings of this study are consistent with several studies showing that SRHC is a tumor suppressor in various cancer types^39–41^.

The marked therapeutic efficacy observed in the SRHC and conjugated nanoparticle groups relative to HCC group underscores the transformative potential of nanoformulations in clinical oncology. By using these specialized carriers, the study demonstrates significant improvements in the bioavailability and site-specific accumulation of therapeutic agents. This targeted approach effectively suppresses the biochemical indicators of malignancy, suggesting that the nano-platform provides a robust shield against systemic degradation, thereby allowing for a more potent interaction with the tumor site. While the current data clearly illustrate the disruption of tumor survival and angiogenic signaling, there remains a critical opportunity to further delineate the precise molecular crosstalk triggered by these interventions. Strengthening the mechanistic depth of these findings would involve a more granular analysis of how the NPs-lncRNA-SRHC interacts with downstream effectors within the intracellular environment. Exploring these pathways in greater detail will clarify whether the observed anti-tumor effects stem from direct gene silencing or a broader modulation of the tumor microenvironment, ultimately providing a more comprehensive understanding of the treatment’s biological impact.

The provided data in Fig. 4 illustrate a clear molecular shift associated with the progression and treatment of Hepatocellular Carcinoma (HCC), primarily driven by the dysregulation of the SENP1/β-catenin/HNF-4α axis. In the HCC group, significant elevations of SENP1 and β-catenin compared with normal controls indicate activation of oncogenic pathways. SENP1 is a deSUMOylating enzyme often implicated in stabilizing proteins that promote cell survival and proliferation. At the same time, β-catenin is a central effector of the Wnt signaling pathway, which facilitates tumor growth and epithelial-mesenchymal transition (EMT)^41,43^. Conversely, the drastic downregulation of HNF-4α in the HCC group reflects a loss of hepatocyte differentiation, as this transcription factor is essential for maintaining the mature liver phenotype.

The lack of a significant difference between the NPs-treated group and the HCC group indicates that the nanoparticles alone do not possess therapeutic properties for these specific gene expressions. Also, treatment with SRHC significantly attenuated the expression of SENP1 and β-catenin while partially restoring HNF-4α, suggesting its potential to inhibit tumor signaling and promote re-differentiation. Moreover, the most pronounced therapeutic effect was observed in the NPs-SRHC-treated group, which showed a significantly greater reduction in oncogenic markers and a higher restoration of HNF-4α than the SRHC-only group. This suggests that the polymer-based delivery system enhances the bioavailability and cellular uptake of SRHC, thereby enabling superior modulation of the underlying molecular pathology^44,45^. Therefore, the data demonstrate that nanoparticle-mediated delivery of SRHC effectively reverses the oncogenic SENP1/β-catenin signature and restores hepatocyte-protective HNF-4α expression, providing a promising strategy for targeted HCC therapy.

The improvement in the hepatic architecture suggests a positive effect of treatment on the HCC. Meanwhile, NPs-SRHC-treated group demonstrated the variable nuclear size of hepatocytes, lymphocytic infiltration, cholestasis, and regression of malignant change, with almost complete restoration of near normal lobular architecture (Fig. 5e). Overall, the microscopic examination of the liver tissue in the experimental groups provides insights into the effects of the different treatments on the HCC. The comparative efficacy of the treatments is evidenced by the degree of architectural restoration. While the SRHC-treated group showed a significant reversal of malignant features, the NPs-SRHC treated group suggests a more nuanced healing process. The presence of lymphocytic infiltration in this group is particularly notable, as it likely represents an active immune-mediated response against the remaining tumor cells^46^.

The profound restoration of hepatic lobular architecture observed in the NPs-SRHC-treated group underscores a critical therapeutic synergy between the lncRNA SRHC and its delivery vehicle. By leveraging a PLGA nanoparticle platform, we successfully bypassed the pharmacokinetic hurdles typically associated with RNA-based therapies, such as rapid systemic clearance and enzymatic degradation. This strategic pairing ensured that the therapeutic cargo achieved the necessary bioavailability and localized concentration required to effectively reprogram the malignant landscape. Rather than focusing on the development of novel polymer chemistries, this study highlights the high-impact utility of a clinically validated carrier to unlock the liver parenchyma’s latent regenerative potential.

Furthermore, the transition from a disorganized malignant phenotype to a structured, regenerative state provides robust evidence that the novelty of this work lies in the regulatory precision of SRHC. When delivered via an optimized nanocarrier, SRHC acts as a potent molecular switch, suppressing oncogenic signaling while simultaneously fostering a microenvironment conducive to healthy tissue repair. The consistency of the histological and biomarker data suggests that the conventional nature of the nanoparticle is a deliberate advantage; it provides a stable, predictable foundation that allows the specific, potent effects of the lncRNA to take center stage. Thus, the significance of this research resides in the successful integration of a reliable delivery system with a novel genetic target to achieve a superior healing response in hepatocellular carcinoma models.

## Strategic pathways for the clinical translation of lncRNA-loaded PLGA nanotherapeutics in hepatocellular carcinoma

For the treatment of hepatocellular carcinoma (HCC), the choice of delivery route is pivotal in determining the therapeutic concentration at the tumor site. While systemic intravenous (IV) injection is the most common approach due to its non-invasive nature, it exposes the nanoparticles to rapid clearance by the mononuclear phagocyte system (MPS), primarily in the spleen and lungs. To enhance clinical applicability, transarterial chemoembolization (TACE) or direct intra-arterial infusion through the hepatic artery presents a highly feasible alternative. By delivering the nanotherapeutic directly into the tumor-feeding vessel, clinicians can achieve a higher local dose while significantly reducing systemic exposure and associated side effects. This strategy aligns well with existing interventional radiology practices for liver cancer.

A primary hurdle in translating lncRNA-nanoparticle research is off-target accumulation. Even with optimized PLGA platforms, a significant fraction of the dose is naturally sequestered in the healthy liver parenchyma and the kidneys, raising concerns regarding long-term organ toxicity. To address this, clinical-stage designs must prioritize surface PEGylation to extend circulation time and the attachment of high-affinity ligands (such as galactosamine or specific antibodies) that recognize overexpressed markers on HCC cells. Ensuring a favorable biodistribution profile requires detailed pharmacokinetic modeling to prove that the lncRNA remains stable within the carrier until it reaches the intracellular environment of the target lesion, preventing premature release that could trigger innate immune responses.

The therapeutic efficacy of lncRNA-based nanomedicines can be significantly amplified when integrated into multimodal treatment regimens. Combining these genetic therapies with standard-of-care kinase inhibitors, such as sorafenib or lenvatinib, may yield synergistic effects by simultaneously inhibiting angiogenic pathways and restoring lncRNA-mediated tumor suppression. Furthermore, as the HCC landscape shifts toward immunotherapy, using nanoparticles to deliver lncRNAs that “warm up” the immunomodulatory microenvironment could sensitize tumors to immune checkpoint inhibitors (e.g., anti-PD-L1). Such combination strategies not only improve objective response rates but also help mitigate the development of drug resistance, offering a more robust framework for personalized HCC management in a clinical setting.

## Conclusion

The findings of this study provide compelling evidence that the administration of the SRHC and its polymerized form (NPs-SRHC) exerts significant anti-tumor effects against hepatocellular carcinoma (HCC). The primary mechanism of action identified is the robust inhibition of cellular proliferation, a hallmark of cancer progression. By demonstrating that SRHC overexpression directly correlates with reduced HCC cell growth, this research highlights SRHC as a critical negative regulator of tumor expansion. Furthermore, the utilization of NPs-SRHC represents a strategic advancement in drug delivery and therapeutic targeting. Its ability to suppress malignancy suggests that polymerization may enhance the stability or bioavailability of the composite, making it a promising therapeutic target for clinical intervention. When compared with the previously established safety profile, in which no systemic toxicity or behavioral distress was observed in vivo, these results position SRHC as a rare candidate that balances high therapeutic efficacy with low biological risk. In summary, this study validates SRHC and NPs-SRHC as potent agents in the fight against liver cancer. These results offer a foundational framework for translating these laboratory findings into clinical practice, potentially offering HCC patients a novel, targeted treatment strategy that prioritizes both tumor reduction and patient safety.

## Data Availability

The data analyzed during the current study available from the corresponding author on reasonable request.

## References

[1] Ferrasi, A. C. et al. New LncRNAs in chronic hepatitis C progression: from fibrosis to hepatocellular carcinoma. *Sci. Rep.* **10**(1), 9886 (2020). 32555359 10.1038/s41598-020-66881-2 PMC7303194

[2] Elmoslemany, A. M., Elzallat, M., Abd-Elfatah, M. H., Mohammed, D. M. & Abd Elhady, E. E. Possible therapeutic effect of frankincense (*Gum olibanum*) and myrrh (*Commiphora myrrha*) resins extracts on DEN/CCL4 induced hepatocellular carcinoma in rats. *Phytomedicine Plus* **4**(1), 100517 (2024).

[3] Wang, Q. B. et al. Tumor compression of the hepatic or portal vein predicts the presence of microvascular invasion and satellite nodules in hepatocellular carcinoma: A retrospective study. *J. Hepatocell. Carcinoma*. **12**, 2055–2067 (2025). 40963958 10.2147/JHC.S544589 PMC12439820

[4] Luo, W. L. et al. Impact of middle hepatic vein resection during hemihepatectomy on surgical outcomes and long-term prognosis in hepatocellular carcinoma: A retrospective study. *J. Hepatocell. Carcinoma*. **12**, 2681–2692 (2025). 41383360 10.2147/JHC.S556306 PMC12689434

[5] Wali, A. F. et al. Epigenetic alterations in hepatocellular carcinoma: Mechanisms, biomarkers, and therapeutic implications. *Pharmaceuticals* **18**(9), 1281 (2025). 41011154 10.3390/ph18091281 PMC12473062

[6] Márton, É. et al. Non-Coding RNAs in cancer: Structure, function, and clinical application. *Cancers* **17**(4), 579 (2025). 40002172 10.3390/cancers17040579 PMC11853212

[7] Wang, Y. & Deng, B. Hepatocellular carcinoma: Molecular mechanism, targeted therapy, and biomarkers. *Cancer Metastasis Rev.* **42**(3), 629–652 (2023). 36729264 10.1007/s10555-023-10084-4

[8] Norollahi, S. E. et al. Analytical and therapeutic profiles of DNA methylation alterations in cancer; an overview of changes in chromatin arrangement and alterations in histone surfaces. *Horm. Mol. Biol Clin. Investig.* **44**(3), 337–356 (2023). 36799246 10.1515/hmbci-2022-0043

[9] Samban, S. S. et al. An insight into the role of alpha-fetoprotein (AFP) in the development and progression of hepatocellular carcinoma. *Mol. Biotechnol.* **66**(10), 2697–2709 (2024). 37782430 10.1007/s12033-023-00890-0

[10] Sang, L. et al. The role of hepatocyte nuclear factor 4α (HNF4α) in tumorigenesis. *Front. Oncol.* **12**, 1011230 (2022). 36249028 10.3389/fonc.2022.1011230 PMC9554155

[11] Ju, L. et al. CircGNAO1 suppresses hepatocellular carcinoma progression and metastasis via sponging miR-182-5p and regulating FOXO1 expression. *Int. Immunopharmacol.* **140**, 112873 (2024). 39098231 10.1016/j.intimp.2024.112873

[12] Cheng, L. et al. LncRNA2Target v2.0: A comprehensive database for target genes of lncRNAs in human and mouse. *Nucleic Acids Res.* **47**(D1), D140–D144 (2019). 30380072 10.1093/nar/gky1051 PMC6323902

[13] Wang, J., Zhang, X., Chen, W., Li, J. & Liu, C. CRlncRNA: A manually curated database of cancer-related long non-coding RNAs with experimental proof of functions on clinicopathological and molecular features. *BMC Med. Genom.* **11**(Suppl 6), 114 (2018). 10.1186/s12920-018-0430-2 PMC6311896 30598113

[14] Alanazi, J. S. et al. MicroRNA-539-5p-Loaded PLGA nanoparticles grafted with iRGD as a targeting treatment for choroidal neovascularization. *Pharmaceutics* **14**(2), 243 (2022). 35213977 10.3390/pharmaceutics14020243 PMC8877575

[15] Ezzat, A., Abdelhamid, A. O., Awady, E., Azeem, M. K. A. E., Mohammed, D. M. & A. S., & The biochemical effects of nano tamoxifen and some bioactive components in experimental breast cancer. *Biomed. Pharmacother.* **95**, 571–576 (2017). 28869895 10.1016/j.biopha.2017.08.099

[16] Reeves, P. G., Nielsen, F. H. & Fahey, G. C. Jr AIN-93 purified diets for laboratory rodents: final report of the American Institute of Nutrition ad hoc writing committee on the reformulation of the AIN-76A rodent diet. *J. Nutr.* **123**(11), 1939–1951 (1993). 8229312 10.1093/jn/123.11.1939

[17] El-Ahwany, E. et al. MicroRNA-195 vector influence on the development of gradually induced hepatocellular carcinoma in murine model. *Ultrastruct. Pathol.* **44**(2), 203–210 (2020). 32216509 10.1080/01913123.2020.1744783

[18] Mohammed, D. M. et al. Bioactive *Moringa oleifera* and *Nigella sativa* oils microcapsules alleviate high-fat-diet induced hepatic oxidative damage and inflammation in rats. *Food Biosci.* **64**, 105873 (2025).

[19] Zhang, W. et al. SENP1 regulates hepatocyte growth factor-induced migration and epithelial-mesenchymal transition of hepatocellular carcinoma. *Tumor Biol.* **37**(6), 7741–7748 (2016). 26695141 10.1007/s13277-015-4406-y

[20] Deldar Abad Paskeh, M., Mirzaei, S., Ashrafizadeh, M., Zarrabi, A. & Sethi, G. Wnt/β-catenin signaling as a driver of hepatocellular carcinoma progression: An emphasis on molecular pathways. *J. Hepatocellular Carcinoma*. **8**, 1415–1444 (2021). 34858888 10.2147/JHC.S336858 PMC8630469

[21] Elshibani, F. A. et al. Investigating the hepatoprotective effects of Onopordum cyrenaicum: Phytochemical, biological, and computational approaches to combat liver damage. *Nat. Prod. Res.* **2**, 1–8 (2025). 10.1080/14786419.2025.2609189 41481366

[22] Kumar, S., Gonzalez, E. A., Rameshwar, P. & Etchegaray, J. P. Non-coding RNAs as mediators of epigenetic changes in malignancies. *Cancers* **12**(12), 3657 (2020). 33291485 10.3390/cancers12123657 PMC7762117

[23] Rubatto, M. et al. Exploring the role of epigenetic alterations and non-coding RNAs in melanoma pathogenesis and therapeutic strategies. *Melanoma Res.* **33**(6), 462–474 (2023). 37788101 10.1097/CMR.0000000000000926

[24] Pi, Y. N., Qi, W. C., Xia, B. R., Lou, G. & Jin, W. L. Long non-coding RNAs in the tumor immune microenvironment: biological properties and therapeutic potential. *Front. Immunol.* **12**, 697083 (2021). 34295338 10.3389/fimmu.2021.697083 PMC8290853

[25] Salem, M. B., Mohammed, D. M., Hammam, O. A. & Elzallat, M. Mitigation of intrahepatic cholestasis induced by 17α-ethinylestradiol via nanoformulation of Silybum marianum L. *BMC Complement. Med. Ther.* **24**(1), 51 (2024). 38263002 10.1186/s12906-024-04351-2 PMC10804614

[26] Soliman, T. N., Karam-Allah, A. A. K., Abo-Zaid, E. M. & Mohammed, D. M. Efficacy of nanoencapsulated *Moringa oleifera* L. seeds and *Ocimum tenuiflorum* L. leaves extracts incorporated in functional soft cheese on streptozotocin-induced diabetic rats. *Phytomedicine Plus* **4**(3), 100598 (2024).

[27] Ezzat, R., Eltabbakh, M. & Kassas, E. M. Unique situation of hepatocellular carcinoma in Egypt: A review of epidemiology and control measures. *World J. Gastrointest. Oncol.* **13**(12), 1919. (2021). 10.4251/wjgo.v13.i12.1919 PMC8713321 35070033

[28] Chen, X. M. et al. ZG16B: A key regulator of tumor progression and immune microenvironment modulation in cancer. *Int. J. Mol. Med.* **57**(3), 58 (2026). 41543187 10.3892/ijmm.2026.5729 PMC12810941

[29] Papaconstantinou, D., Tsilimigras, D. I. & Pawlik, T. M. Recurrent hepatocellular carcinoma: Patterns, detection, staging and treatment. *J. Hepatocell. Carcinoma* **9**, 947–957 (2022). 36090786 10.2147/JHC.S342266 PMC9450909

[30] Nevola, R. et al. Predictors of early and late hepatocellular carcinoma recurrence. *World J. Gastroenterol.* **29**(8), 1243 (2023). 36925456 10.3748/wjg.v29.i8.1243 PMC10011963

[31] Samban, S. S. et al. An insight into the role of alpha-fetoprotein (AFP) in the development and progression of hepatocellular carcinoma. *Mol. Biotechnol.* **66**(10), 2697–2709 (2024). 37782430 10.1007/s12033-023-00890-0

[32] Mohammed, D. M., Salem, M. B., Elzallat, M., Hammam, O. A. & Suliman, A. A. *Moringa oleifera* L. mediated zinc oxide nano-biofertilizer alleviates non-alcoholic steatohepatitis via modulating de novo lipogenesis pathway and miRNA-122 expression. *Food Bioscience*. **60**, 104286 (2024).

[33] Orrapin, S. et al. Clinical implication of circulating tumor cells expressing epithelial mesenchymal transition (EMT) and cancer stem cell (CSC) markers and their perspective in HCC: A systematic review. *Cancers* **14**(14), 3373 (2022). 35884432 10.3390/cancers14143373 PMC9322939

[34] Pathak, A., Pal, A. K., Roy, S., Nandave, M. & Jain, K. Role of angiogenesis and its biomarkers in development of targeted tumor therapies. *Stem Cells Int.* **2024**(1), 9077926 (2024). 38213742 10.1155/2024/9077926 PMC10783989

[35] Abou Baker, D. H. & Mohammed, D. M. Polyphenolic rich fraction of *Physalis peruviana* calyces and its nano emulsion induce apoptosis by caspase 3 up-regulation and G2/M arrest in hepatocellular carcinoma. *Food Biosci.* **50**, 102007 (2022).

[36] Lee, C. C. et al. Epithelial cell adhesion molecule (EpCAM) regulates HGFR signaling to promote colon cancer progression and metastasis. *J. Transl Med.* **21**(1), 530 (2023). 37543570 10.1186/s12967-023-04390-2 PMC10404369

[37] El-Kholy, M. A. et al. The role of epithelial cell adhesion molecule cancer stem cell marker in evaluation of hepatocellular carcinoma. *Medicina* **60**(6), 915 (2024). 38929532 10.3390/medicina60060915 PMC11205386

[38] Chang, C. H. et al. EpCAM signaling in oral cancer stem cells: Implications for metastasis, tumorigenicity, and therapeutic strategies. *Curr. Issues Mol. Biol.* **47**(2), 123 (2025). 39996844 10.3390/cimb47020123 PMC11854592

[39] Wang, S. et al. LINC01018 confers a novel tumor suppressor role in hepatocellular carcinoma through sponging microRNA-182-5p. *Am. J. Physiol. Gastrointest. Liver Physiol.* **317**(2), G116–G126 (2019). 10.1152/ajpgi.00005.2019 31021172

[40] Su, H. et al. Long non-coding RNA LINC01018 inhibits human glioma cell proliferation and metastasis by directly targeting miRNA-182-5p. *J. Neuro Oncol.* **160** (1), 67–78 (2022). 10.1007/s11060-022-04113-5 36094613

[41] Luo, W., Li, T., Song, Q., Zhang, L. & Cao, M. Prognostic value of lncRNA LINC01018 in prostate cancer by regulating miR-182-5p (the role of LINC01018 in prostate cancer). *Nucleosides Nucleotides Nucleic Acids* **43** (10), 1077–1089 (2024). 38147366 10.1080/15257770.2023.2298408

[42] Alsaab, H. O. Pathological role of long non-coding (lnc) RNA in the regulation of Wnt/β-catenin signaling pathway during epithelial-mesenchymal transition (EMT). *Pathol. Res. Pract.* **248**, 154566 (2023). 10.1016/j.prp.2023.154566 37285735

[43] Xue, W. et al. Wnt/β-catenin-driven EMT regulation in human cancers. *Cell. Mol. Life Sci.* **81** (1), 79 (2024). 38334836 10.1007/s00018-023-05099-7 PMC10857981

[44] Acharya, B., Behera, A., Behera, S. & Moharana, S. Recent advances in nanotechnology-based drug delivery systems for the diagnosis and treatment of reproductive disorders. *ACS Appl. Bio Mater.* **7**(3), 1336–1361 (2024). 38412066 10.1021/acsabm.3c01064

[45] Ullah, A. et al. Advancing therapeutic strategies with polymeric drug conjugates for nucleic acid delivery and treatment. *Int. J. Nanomed.* **20**, 25–52 (2025). 10.2147/IJN.S429279 PMC11717654 39802382

[46] Playoust, E., Remark, R., Vivier, E. & Milpied, P. Germinal center-dependent and-independent immune responses of tumor-infiltrating B cells in human cancers. *Cell Mol. Immunol.* **20**(9), 1040–1050 (2023). 37419983 10.1038/s41423-023-01060-7 PMC10468534

